# Determinants and Effectiveness of Extending the Duration of Adjuvant Hormone Therapy beyond 5 Years in Patients with Breast Cancer

**DOI:** 10.1158/0008-5472.CAN-22-0900

**Published:** 2022-08-18

**Authors:** Erwei Zeng, Wei He, Arvid Sjölander, Jenny Bergqvist, Kamila Czene

**Affiliations:** 1 Department of Medical Epidemiology and Biostatistics, Karolinska Institutet, Stockholm, Sweden.; 2 Chronic Disease Research Institute, The Children's Hospital, and National Clinical Research Center for Child Health, School of Public Health, School of Medicine, Zhejiang University, Hangzhou, Zhejiang, China.; 3 Department of Nutrition and Food Hygiene, School of Public Health, Zhejiang University, Hangzhou, Zhejiang, China.

## Abstract

Clinical guidelines have recommended patients with high-risk breast cancer to extend adjuvant hormone therapy beyond 5 years. However, the prevalence, predictors, and outcomes of extended adjuvant hormone therapy in the real world remain unknown. By linking six Swedish health registries, we prospectively followed 13,168 patients with breast cancer (2005–2020) from their first prescription of tamoxifen or aromatase inhibitors and categorized them as extending or not extending adjuvant hormone therapy. Cox regression analysis was used to investigate whether extended therapy was associated with breast cancer outcomes. Among patients with breast cancer who were recommended to extend adjuvant hormone therapy by the national guidelines, the proportion of women who extended therapy increased 5 folds during the past 10 years, reaching 80.9% during 2018 to 2020. Patients were more likely to extend therapy after completing 5-year adjuvant hormone therapy if they were young [40 vs. ≥65 years: OR, 1.71; 95% confidence interval (CI), 1.13–2.58], had positive lymph nodes (OR, 2.25; 95% CI, 1.85–2.73), had high tumor grade (grade 3 vs. 1: OR, 1.79; 95% CI, 1.34–2.39), received chemotherapy (OR, 5.22; 95% CI, 4.19–6.50), had first-degree relatives who died from breast cancer (OR, 1.84; 95% CI, 1.21–2.81), or had a high income (OR, 1.23; 95% CI, 1.01–1.49). Extended use of adjuvant hormone therapy was statistically significantly associated with improved disease-free survival (HR, 0.72; 95 CI%, 0.55–0.95). This study provides real-world evidence showing the use and improved breast cancer outcomes of extended adjuvant hormone therapy beyond 5 years.

**Significance:**

The proportion of patients with breast cancer extending adjuvant hormone therapy beyond 5 years has increased dramatically in recent years, which is associated with improved patient outcomes.

## Introduction

Adjuvant hormone therapy for 5 years has been the standard of care for patients with estrogen receptor–positive breast cancer in the past several decades. However, after finishing the standard 5 years of adjuvant hormone therapy, some patients still have a high risk of distant recurrence during 15 years of follow-up [from 13% to 41% depending on tumor stage (1–3)]. Therefore, an extended duration of adjuvant hormone therapy beyond 5 years may be needed to further improve breast cancer outcomes.

Clinical trials have linked extended duration of adjuvant hormone therapy with better breast cancer outcomes (4–10), with a greater effect size among women with lymph node–positive tumors, larger tumor size, or chemotherapy (11). Consequently, current clinical guidelines recommend an extended duration of tamoxifen beyond 5 years for patients with high-risk breast cancer recurrence (e.g., patients with positive lymph nodes; refs. 12–14). However, despite these recommendations, the actual use of extended therapy in the real world remains unknown. Furthermore, whether extending aromatase inhibitors for patients who use aromatase inhibitors or switched therapy in the first 5 years is still in debate (15–18), which have led to inconsistent recommendations in clinical guidelines (12–14).

Using data from several Swedish health registers, this population-based study aimed to (i) examine the prevalence of extended adjuvant hormone therapy and how it changed over time; (ii) identify clinical characteristics and patients’ factors that determine the use of extended adjuvant hormone therapy; (iii) investigate the association between extended adjuvant hormone therapy and survival outcomes in a real-world setting.

## Patients and Methods

### Data source and study population

The Regional Ethical Review Board in Stockholm, Sweden, approved the study. The Stockholm-Gotland Quality Register for Breast Cancer (the Stockholm-Gotland Breast Cancer Register, 1976–2007, and the National Quality Register for Breast Cancer, 2008 onwards) hold detailed information on tumor characteristics and treatments for all patients with breast cancer diagnosed in the Stockholm-Gotland region since 1976 (19, 20). The Swedish Prescribed Drug Register records all dispensed prescribed drugs in pharmacies nationwide since July 2005 (21). Both registers are of high quality, with over 99% completeness (19–21). The Swedish Cause-of-Death Register includes data on the date and cause of deaths in Sweden since 1952, with 96% completeness on the underlying cause of deaths (22). The Swedish Multi-Generation Register consists of all Swedish residents born after 1931 with links between children and parents, and is considered as a completed database from 1991 (23). The Swedish Longitudinal Integrated Database for Health Insurance and Labor Market Studies collects data on socioeconomic status, including education and income (24).

Using the unique Personal Identification Numbers (25), we linked the Stockholm-Gotland Quality Register for Breast Cancer to the Swedish Prescribed Drug Register. Through this linkage, we identified 13,511 women diagnosed with estrogen receptor–positive breast cancer at age below 75 between 2005 and 2020, who initiated adjuvant hormone therapy [≥ two prescriptions of tamoxifen (ATC code L02BA01) and/or aromatase inhibitors (ATC code L02BG)], in Stockholm, Sweden. We excluded 152 patients with distant metastases at diagnosis, 158 patients who initiated adjuvant hormone therapy over 1 year after a breast cancer diagnosis, and 33 patients who had any breast cancer event [local recurrence, distant metastasis, or contralateral breast cancers (>3 months after the primary breast cancer)] before initiating therapy, leaving 13,168 patients in the final cohort.

### Discontinuation of adjuvant hormone therapy

Discontinuation of adjuvant hormone therapy was defined as having intervals between any two consecutive refills of tamoxifen or aromatase inhibitors exceeding 6 months during the first 5 years (26). In Sweden, a 3-month supply of prescription drugs is the maximum that can be dispensed. Therefore, a gap of 6-month indicates that ≥2 dispenses have been missed, thus resulting in a shortage of the drug.

### Extension of adjuvant hormone therapy

Among patients who finished the 5-year adjuvant hormone therapy and remained free of recurrence at the end of 5-year therapy, we defined whether the patients extended their therapy or not. Specifically, extended therapy was defined as continuing the therapy for ≥6 months and filling ≥2 prescriptions of tamoxifen or aromatase inhibitors beyond the 5-year adjuvant hormone therapy. Information on the prescription of tamoxifen or aromatase inhibitors was obtained from the Swedish Prescribed Drug Register.

### Predictors for extended adjuvant hormone therapy

Information on age, tumor size, lymph nodes status, tumor grade, progesterone receptor status, HER2 status at diagnosis, and treatments was retrieved from the Stockholm-Gotland Quality Register for Breast Cancer. Information on therapy type at baseline was retrieved from the Swedish Prescribed Drug Register. Family history of breast cancer or death from breast cancer at diagnosis was obtained by linking the Multi-Generation Register to the Swedish Cancer Register and the Cause-of-Death Register. Socioeconomic status at diagnosis, including education and personal income, was retrieved from the Swedish Longitudinal Integrated Database for Health Insurance and Labor Market Studies. Income was measured using average 5-year income before diagnosis and categorized into three groups according to tertiles.

### Outcomes

Disease-free survival was defined as time to local recurrence, distant metastasis, contralateral breast cancer, second primary cancer (excluding skin cancer), or death from any cause. We also considered overall survival, i.e., time to death from any cause. Information on distant metastasis, local recurrence, and contralateral breast cancer was retrieved from the Stockholm-Gotland Quality Register for Breast Cancer. Information on second primary cancer was retrieved from the Swedish Cancer Register. Information on the death was retrieved from the Swedish Cause-of-Death Register.

### Follow-up

#### Follow-up to define treatment discontinuation during the first 5 years

Patients were followed from the first prescription of tamoxifen or aromatase inhibitors until local recurrence, distant metastasis, contralateral breast cancer, death, emigration, completion of 5-year treatment or end of the study period (August 31, 2020), whichever came first, to define treatment discontinuation.

#### Follow-up to define extended adjuvant hormone therapy beyond 5 years

Among patients who continued adjuvant hormone therapy in the first 5 years, patients were followed from the end of 5 years to 5.5 years to define extended adjuvant hormone therapy.

#### Follow-up to define disease-free survival and overall survival

Patients were followed from the end of 5.5 years since treatment initiation until death, emigration or end of the study period (September 17, 2020), whichever came first, to define the event for the corresponding survival analysis.

### Statistical analysis

Kaplan–Meier analysis was performed to estimate the cumulative rate of continued use of adjuvant hormone therapy over 10 years. We used age-adjusted and multivariable-adjusted logistic regression to identify predictors for extended adjuvant hormone therapy. Kaplan–Meier analysis was used to estimate rate of disease-free survival and overall survival among patients who extended adjuvant hormone therapy and those who did not. Cox regression model was used to investigate the association of extended adjuvant hormone therapy with disease-free survival and overall survival, adjusting for age at diagnosis, calendar period of cancer diagnosis, tumor size, lymph node status, tumor grade, progesterone receptor status, chemotherapy, radiotherapy, Charlson Comorbidity Index at therapy extension, education and income at diagnosis. We conducted subgroup analyses by repeating the Cox regression analyses within strata defined by tumor size, lymph node status, tumor grade and type of adjuvant hormone therapy at baseline.

All analyses were performed using SAS software (v 9.4; SAS Institute; RRID:SCR_008567) and Stata software (v 15.1; Stata Corporation; RRID:SCR_012763), at a two-tailed alpha level of 0.05.

### Data availability

Data underlying this article were provided by the Socialstyrelsen and Statistics Sweden by permission, and cannot be shared publicly due to the Swedish Secrecy Act. Researchers may apply for data from the Socialstyrelsen, and the Statistics Sweden according to Swedish law.

## Results

### Use of adjuvant hormone therapy across 10 years

**Figure 1.**
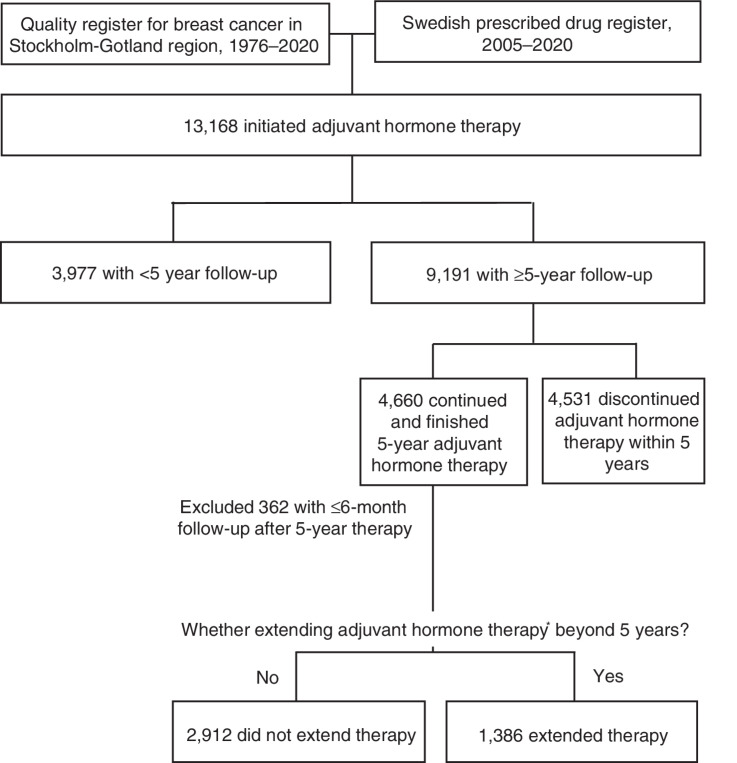
Flow chart of study population. Note: Extending adjuvant hormone therapy was defined as continuing the therapy for ≥6 months and filling ≥2 prescriptions of tamoxifen or aromatase inhibitors beyond the 5-year adjuvant hormone therapy.

**Figure 2.**
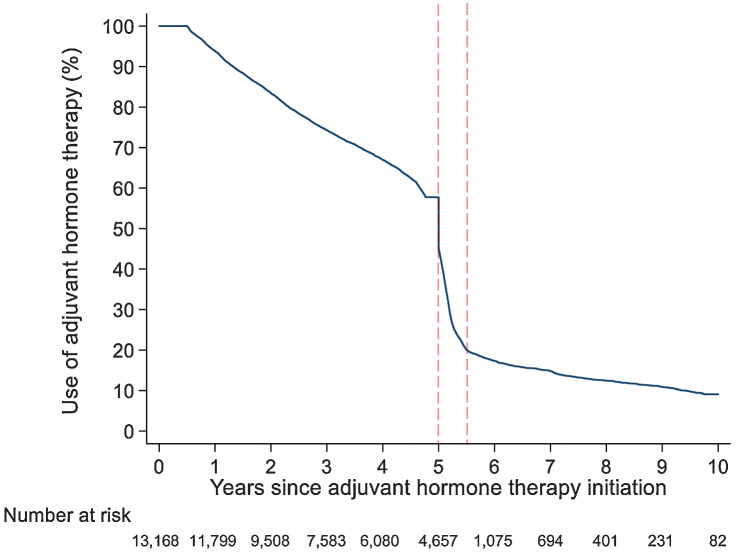
Use of adjuvant hormone therapy across 10 years in women diagnosed with breast cancer in Stockholm, Sweden, 2005–2020. The dashed vertical lines indicate six months after finishing 5 years of adjuvant hormone therapy—a period that was used to define therapy extension.

Among 4,660 patients who finished the 5-year adjuvant hormone therapy, 1,386 (29.7%) patients extended their therapy (Fig. 1). Consistently, the cumulative rate of continued use of adjuvant hormone therapy decreased sharply from 57.7% to 20.0% within six months after finishing the first five years of adjuvant hormone therapy (Fig. 2).

### Prevalence of extended adjuvant hormone therapy

**Figure 3.**
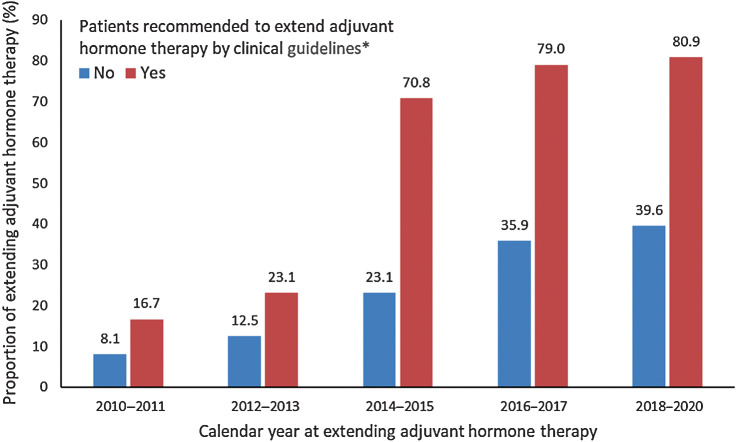
Prevalence of extended therapy among patients who finished 5-year adjuvant hormone therapy, by whether recommended to extend therapy by the Swedish National Clinical Guideline, 2010 to 2020. Note: 365 of 504 patients who met the criteria to extend adjuvant hormone therapy in the clinical guideline extended their therapy after finishing 5-year adjuvant hormone therapy, and 993 of 3,727 patients who did not meet therapy extension criteria extended their therapy after finishing 5-year adjuvant hormone therapy. *, Patients with the following characteristics are recommended to extend adjuvant hormone therapy beyond 5 years by the Swedish National Clinical Guideline: during 2010 to 2014, postmenopausal women with lymph node–positive tumor and treated with tamoxifen during the first 5 years, which is consistent with ASCO guideline 2010 and ESMO guideline 2010; during 2015 to 2017, further including premenopause women with lymph node–positive tumor and treated with tamoxifen during the first 5 years, which is consistent with ASCO guideline 2014 and ESMO guideline 2015; during 2018 to 2020, further including postmenopause women with lymph node–positive tumor and treated with aromatase inhibitors during the first 5 years, which is consistent with ASCO guideline 2018.

Figure 3 shows that the prevalence of extended adjuvant hormone therapy over the last 10 years. In the past decade, the proportion of patients who extended their therapy increased dramatically. Among patients who met the criteria for therapy extension by the clinical guideline (14, 27, 28), the prevalence increased from 16.7% during 2010 to 2011 to 80.9% during 2018 to 2020. Among patients who did not met the extension criteria, the prevalence of extended therapy also increased by almost 5 times, from 8.1% during 2010 to 2011 to 39.6% during 2018 to 2020.

### Predictors for extended adjuvant hormone therapy

Table 1 presents predictors for extended adjuvant hormone therapy. Younger age at diagnosis, use of aromatase inhibitors (versus tamoxifen), larger tumor size, positive lymph node involvement, higher tumor grade, negative progesterone receptor status, positive HER2 status, chemotherapy, radiotherapy, having first-degree relatives dying from breast cancer, higher income was associated with a higher likelihood to extend therapy after finishing their 5-year adjuvant hormone therapy in the age-adjusted model. In the multivariable-adjusted model, younger age at diagnosis (<40 vs. ≥65 years: OR, 1.71; 95% confidence interval (CI), 1.13–2.58), positive lymph node involvement (OR, 2.25; 95% CI, 1.85–2.73), higher tumor grade (grade 3 vs. 1: OR, 1.79; 95% CI, 1.34–2.39), chemotherapy (OR, 5.22; 95% CI, 4.19–6.50), having first-degree relatives dying from breast cancer (OR, 1.84; 95% CI, 1.21–2.81), high income (OR, 1.23; 95% CI, 1.01–1.49) remained statistically significant.

**Table 1.** Clinical characteristics and their relationship with extending therapy after finishing 5-years adjuvant hormone therapy in women diagnosed with breast cancer in Stockholm, Sweden, 2005–2020.

			OR (95% CI)	
Characteristics	Total	Extended therapy^a^, (%)	Age-adjusted	Multivariable^b^
Age at diagnosis (years)				
<40	155	87 (56.1)	3.81 (2.71–5.36)	1.71 (1.13–2.58)
40–49	848	384 (45.3)	2.46 (2.05–2.96)	1.72 (1.36–2.17)
50–64	2,002	590 (29.5)	1.24 (1.06–1.46)	1.01 (0.83–1.23)
≥65	1,293	325 (25.1)	1.00 (reference)	1.00 (reference)
Therapy type at baseline				
Tamoxifen	2,636	683 (25.9)	1.00 (reference)	1.00 (reference)
Aromatase inhibitors	1,662	703 (42.3)	3.54 (3.03–4.13)	1.13 (0.93–1.38)
Tumor size (mm)				
≤20	2,768	716 (25.9)	1.00 (reference)	1.00 (reference)
>20	1,517	665 (43.8)	2.13 (1.86–2.44)	1.03 (0.87–1.23)
Lymph node involvement				
Negative	2,896	628 (21.7)	1.00 (reference)	1.00 (reference)
Positive	1,335	730 (54.7)	4.21 (3.66–4.86)	2.25 (1.85–2.73)
Elston–Ellis tumor grade				
1	920	139 (15.1)	1.00 (reference)	1.00 (reference)
2	2,191	603 (27.5)	2.13 (1.73–2.61)	1.37 (1.08–1.74)
3	842	428 (50.8)	5.36 (4.27–6.74)	1.79 (1.34–2.39)
Progesterone receptor status				
Positive	3,523	1,112 (31.6)	1.00 (reference)	1.00 (reference)
Negative	696	234 (33.6)	1.22 (1.02–1.46)	1.01 (0.81–1.25)
HER2 status^c^				
Negative	3,216	1,078 (33.5)	1.00 (reference)	1.00 (reference)
Positive	335	196 (58.5)	2.46 (1.94–3.11)	1.03 (0.78–1.36)
Chemotherapy				
No	2,457	311 (12.7)	1.00 (reference)	1.00 (reference)
Yes	1,789	1,057 (59.1)	9.68 (8.27–11.34)	5.22 (4.19–6.50)
Radiotherapy				
No	577	159 (27.6)	1.00 (reference)	1.00 (reference)
Yes	3,673	1,206 (32.8)	1.36 (1.12–1.67)	0.85 (0.67–1.08)
Breast cancer among female first-degree relatives				
No	3,371	1,068 (31.7)	1.00 (reference)	1.00 (reference)
Yes	619	210 (33.9)	1.16 (0.96–1.39)	1.16 (0.93–1.45)
Death from breast cancer among female first-degree relatives				
No	3,844	1,218 (31.7)	1.00 (reference)	1.00 (reference)
Yes	146	60 (41.1)	1.66 (1.18–2.34)	1.84 (1.21–2.81)
Education (years)				
≤9	623	172 (27.6)	1.00 (reference)	1.00 (reference)
9–12	1,688	527 (31.2)	1.07 (0.87–1.32)	1.10 (0.85–1.41)
>12	1,958	674 (34.4)	1.20 (0.98–1.47)	1.24 (0.97–1.58)
Income				
Low	1,432	419 (29.3)	1.00 (reference)	1.00 (reference)
Middle	1,433	427 (29.8)	0.94 (0.80–1.11)	0.85 (0.69–1.03)
High	1,433	540 (37.7)	1.37 (1.16–1.61)	1.23 (1.01–1.49)

^a^Extended adjuvant hormone therapy was a binary outcome, which was defined as continuing the therapy for ≥6 months and filling ≥2 prescriptions of tamoxifen or aromatase inhibitors beyond the 5-year adjuvant hormone therapy.

^b^Adjusted for age at diagnosis, calendar period of cancer diagnosis, tumor size, lymph node status, tumor grade, progesterone receptor status, chemotherapy, and radiotherapy.

^c^HER2 status was recorded from 2007.

### Extended adjuvant hormone therapy and survival outcomes

**Figure 4.**
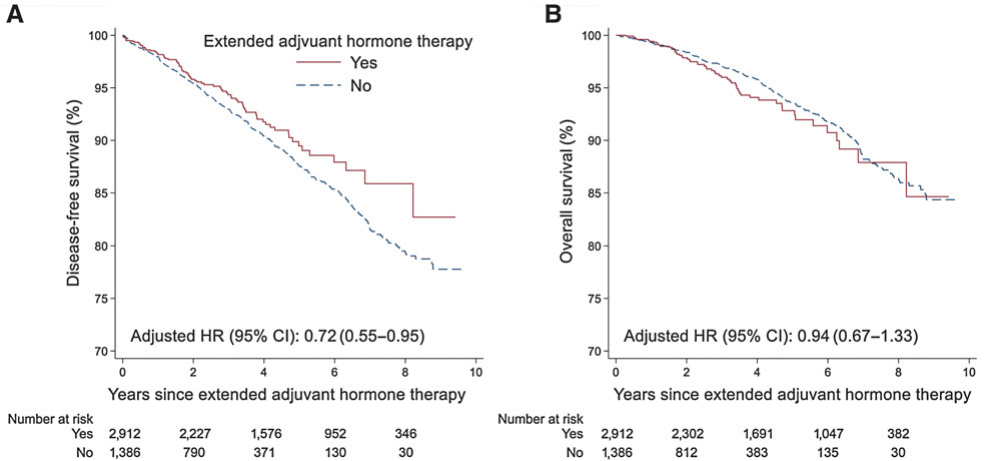
Disease-free survival and overall survival in patients who extended therapy and those who did not. **A** and **B,** Disease-free survival (**A**) and overall survival (**B**). Note: HR was adjusted for age at diagnosis, calendar period of cancer diagnosis, tumor size, lymph node status, tumor grade, progesterone receptor status, chemotherapy, radiotherapy, Charlson Comorbidity Index at extension, education, and income at diagnosis.

Figure 4 shows disease-free survival and overall survival among patients who extended adjuvant hormone therapy and those who did not. The incidence of events for disease-free survival analysis was 26.8 (95% CI, 24.2–29.8) per 1,000 person-years among patients who did not extend adjuvant hormone therapy, and 20.6 (95% CI, 16.6–25.6) per 1,000 person-years among patients who did extend their therapy after finishing 5-year adjuvant hormone therapy (Supplementary Table S1). Cox regression analysis showed an association of the extended duration of adjuvant hormone therapy with improved disease-free survival (HR for occurrence of disease-free survival events, 0.72; 95 CI%, 0.55–0.95). However, no statistically significant difference was found for overall survival when comparing extenders versus non-extenders of adjuvant hormone therapy (HR, 0.94; 95 CI%, 0.67–1.33). Subgroup analyses by tumor size, lymph node status, tumor grade or type of adjuvant hormone therapy at baseline showed consistent results (Supplementary Table S2).

## Discussion

This population-based study investigated the use of extended adjuvant hormone therapy beyond 5 years in clinical practice. We found that the proportion of women who extended adjuvant hormone therapy increased 5 times in the last decades, both among those who met and those who did not met the extension criteria in clinical guidelines. Patients with worse tumor characteristics (positive lymph nodes, high tumor grade), receiving chemotherapy, or having first-degree relatives dying from breast cancer were more likely to extend their adjuvant hormone therapy beyond 5 years. Extended adjuvant hormone therapy was associated with better disease-free survival.

Tamoxifen users with positive lymph nodes have been recommended to receive extended therapy since 2010 (28–30). In our study, nearly 90% of tamoxifen users with positive lymph nodes received extended therapy in 2019, indicating relatively good adherence to clinical guidelines. However, why another 10% of patients with breast cancer did not extend their therapy still needs further investigation to make sure that all eligible women can get access to, and benefit from, potentially life-saving medications.

Aromatase inhibitors users have been recommended to use extended therapy since 2018 (12, 14). However, in 2017, 71% of patients with lymph node–positive disease extended their therapy after 5-year aromatase inhibitors. This finding is unexpected but reasonable since around 2017, emergent clinical trials have published data on extended aromatase inhibitors (15–18). This indicates that there is a time lag between accumulating evidence and clinical guidelines, and clinicians may have already prescribed extended therapy to aromatase inhibitors users based on their beliefs and knowledge.

Patients with breast cancer with chemotherapy were over 5 times more likely to receive extended adjuvant hormone therapy than those without chemotherapy in our study. Receiving chemotherapy reflects worse tumor characteristics, such as positive lymph nodes, large tumor size, high proliferation rate and/or positive HER2 status (31). This result is consistent with the current clinical guidelines that recommend only patients with high recurrence risk to receive extended adjuvant hormone therapy (12–14).

Patients with breast cancer who had first-degree relatives dying from breast cancer had 84% higher odds of extending adjuvant hormone therapy beyond 5 years. These patients are generally expected to be highly motivated and thus may prefer to extend their therapy when offered. Furthermore, clinicians may also take the family history of death from breast cancer into account, considering the previous studies showing that breast cancer survival is associated among relatives (32–34). Extension rates were higher among younger women in agreement with worse prognosis in these women (35, 36). High education and income were associated with 24% and 23% higher odds of extending extend adjuvant hormone therapy, respectively. Overall, our findings suggests that both disease severity and patient characteristics may influence the decision to extend adjuvant hormone therapy.

Our study provides real-world evidence showing that extended adjuvant hormone therapy beyond 5 years may improve breast cancer outcomes. We observed a 28% improvement in disease-free survival among patients who extended therapy, which was comparable to the effects observed in clinical trials (4–9, 15–18). Our study also showed that extended therapy was associated with improved disease-free survival among patients who finished 5-year aromatase inhibitors therapy. This finding has great clinical implications because the existing clinical guidelines have made contradictory recommendations regarding extending adjuvant hormone therapy among patients using aromatase inhibitors in the first 5 years (12–14).

While extended adjuvant hormone therapy is associated with reduced risk for breast cancer events, it may also increase the risk of other diseases such as cardiovascular diseases and bone fractures (37). Therefore, extended adjuvant hormone therapy may not be recommended to all patients with breast cancer. In our study, after adjusting for tumor characteristics, treatments, comorbidity, and other potential confounders such as patients’ education and income, extended adjuvant hormone therapy was not significantly associated with overall survival.

The following limitations in our study should be mentioned. First, the association between extended adjuvant hormone therapy and breast cancer outcomes may be confounded by indication, because patients with worse tumor characteristics were more likely to extend therapy. However, this confounding would likely attenuate rather than amplify the observed association. We thus view our observed associations as conservative. We also adjusted for tumor characteristics and treatments to minimize its influence. Second, by using the Prescribed Drug Register, patients might only have a refill record but not take the drug. Third, because patients with breast cancer were recommended to extend their adjuvant hormone therapy only in recent years, we were unable to investigate the long-term health effect of extended adjuvant hormone therapy (9).

In summary, the proportion of patients with breast cancer extending their adjuvant hormone therapy beyond 5 years have increased dramatically in recent years, both among patients who meet and those who do not meet the current therapy extension criteria in clinical guidelines. Further studies are needed to determine the factors that can be used to identify patients who are likely to benefit from extended adjuvant hormone therapy.

## Supplementary Material

Supplementary Data
