# Supplementary material for: Determinants and Effectiveness of Extending the Duration of Adjuvant Hormone Therapy beyond 5 Years in Patients with Breast Cancer
Source: Cancer Res. 2022 Aug 18;82(19):3614–21. doi: 10.1158/0008-5472.CAN-22-0900 (PMC9530643; doi:10.1158/0008-5472.CAN-22-0900)
Supplement: Supplementary Data [file can-22-0900_supplementary_data_suppsd.docx]

**Determinants and effectiveness of extending the duration of adjuvant hormone therapy beyond 5 years in breast cancer patients**

**Supplementary Table**

**Table S1.** Extended adjuvant hormone therapy and its association with survival outcomes

| Survival outcomes | Extended therapy | Total | Outcome | Events per 1000 person-years (95%CI) |
| --- | --- | --- | --- | --- |
| **Disease-free survival** | No | 2912 | 350 | 26.8(24.2-29.8) |
|  | Yes | 1386 | 81 | 20.6(16.6-25.6) |
| **Overall survival** | No | 2912 | 205 | 25.5(23.0-28.4) |
|  | Yes | 1386 | 57 | 20.2(16.2-25.1) |

**Table S2.** Hazard ratios and 95% confidence intervals of breast cancer outcomes among patients who finished 5-year adjuvant hormone therapy, comparing patients who extended therapy to those who did not extend therapy, stratified by tumor size, lymph node status, tumor grade or type of adjuvant hormone therapy at baseline

|  | Disease-free survival | |  | All-cause mortality | |  |
| --- | --- | --- | --- | --- | --- | --- |
|  | HR^a^ (95% CI) | P_interaction_ |  | HR^a^ (95% CI) | P_interaction_ |  |
| **Tumor size (mm)** |  | 0.24 |  |  | 0.45 |  |
| ≤ 20 | 0.59(0.38-0.92) |  |  | 0.79(0.47-1.34) |  |  |
| > 20 | 0.82(0.56-1.20) |  |  | 1.06(0.65-1.72) |  |  |
| **Lymph node status** |  | 0.67 |  |  | 0.77 |  |
| Negative | 0.81(0.53-1.25) |  |  | 0.95(0.55-1.65) |  |  |
| Positive | 0.64(0.43-0.95) |  |  | 0.90(0.56-1.44) |  |  |
| **Tumor grade** |  | 0.20 |  |  | 0.83 |  |
| Grade 1 & 2 | 0.69(0.48-1.00) |  |  | 1.04(0.67-1.62) |  |  |
| Grade 3 | 0.81(0.46-1.42) |  |  | 0.93(0.46-1.88) |  |  |
| **Type of adjuvant hormone therapy at baseline** |  | 0.42 |  |  | 0.76 |  |
| Tamoxifen | 0.82(0.54-1.25) |  |  | 1.03(0.60-1.78) |  |  |
| Aromatase inhibitors | 0.66(0.45-0.96) |  |  | 0.89(0.57-1.41) |  |  |

^a^ Hazard ratio (HR) was adjusted for age at diagnosis, calendar period of cancer diagnosis, tumor size, lymph node status, tumor grade, progesterone receptor status, chemotherapy, radiotherapy, Charlson Comorbidity Index at extension, education and income.
